# The Milan Score Predicts Objective Gastroesophageal Reflux Disease in Patients With Type 2 Esophagogastric Junction

**DOI:** 10.1111/nmo.14987

**Published:** 2025-01-06

**Authors:** Davide Ferrari, Stefano Siboni, Marco Sozzi, Pierfrancesco Visaggi, Ivan Kristo, Salvatore Tolone, Elisa Marabotto, Daniele Bernardi, Sebastian F. Schoppmann, Benjamin D. Rogers, Anthony Hobson, Jordan Haworth, Yeong Yeh Lee, Brian E. Louie, Takahiro Masuda, Megan L. Ivy, Pamela Milito, Erica Centorrino, Dimitrios Theodorou, Tania Triantafyllou, Andrea Pasta, Francesco Calabrese, Vincent Tee, Lorenzo Cusmai, Roberto Penagini, Marina Coletta, Edoardo Savarino, Emanuele Asti, C. Prakash Gyawali, Nicola De Bortoli

**Affiliations:** ^1^ Division of General and Emergency Surgery, IRCCS Policlinico San Donato University of Milan San Donato Milanese Italy; ^2^ Division of Gastroenterology University of Pisa Pisa Italy; ^3^ Upper‐GI‐Service, Medizinische Universität Wien Austria; ^4^ Division of General, Mini‐Invasive and Bariatric Surgery Universita degli Studi della Campania, School of Medicine Naples Italy; ^5^ Gastroenterology Unit IRCCS Policlinico San Martino Genoa Italy; ^6^ Division of Gastroenterology Washington University School of Medicine St. Louis Missouri USA; ^7^ The Functional Gut Clinic London UK; ^8^ School of Medical Sciences and GI Function and Motility Unit Universiti Sains Malaysia Kota Bharu Malaysia; ^9^ Division of Thoracic Surgery, Swedish Medical Center Digestive Health Institute Seattle Washington USA; ^10^ Department of Surgery Jikei University School of Medicine Tokyo Japan; ^11^ Foregut Surgery Unit University of Athens School of Medicine Athens Greece; ^12^ Gastroenterology and Endoscopy Unit Fondazione IRCCS Ca' Granda Ospedale Maggiore Policlinico Milan Italy; ^13^ Division of Gastroenterology, Department of Surgical, Oncological and Gastroenterological Sciences University of Padua Padua Italy

**Keywords:** esophago‐gastric junction morphology, gastro‐esophageal reflux disease, hiatal hernia, high‐resolution manometry

## Abstract

**Introduction:**

High‐resolution manometry (HRM) allows assessment of esophagogastric junction (EGJ) disruption. While type 3 EGJ predicts definitive gastroesophageal reflux disease (GERD), type 2 EGJ is less clearly implicated in GERD pathogenesis. This study aimed to characterize physiologic findings in type 2 EGJ to determine if the HRM‐based Milan Score can define GERD within type 2 EGJ.

**Methods:**

535 patients with suspected GERD who underwent HRM and reflux monitoring were retrospectively analyzed. Clinical, HRM, and reflux study data were compared between the EGJ morphology subtypes, with objective GERD defined according to Lyon Consensus 2.0. The Milan Score, a novel metric that integrates ineffective esophageal motility, EGJ‐contractile integral, EGJ morphology, and straight leg raise response, was abnormal when ≥ 137 (risk rate 50% for GERD). Receiver operating characteristic (ROC) curve analysis was performed to assess the accuracy of the Milan Score to predict objective GERD.

**Results:**

Type 3 EGJ was associated with the highest rate of objective GERD, followed by type 2 and type 1 EGJ (*p* < 0.001), with a corresponding stepwise increase in AET from type 1 to 3 EGJ (p < 0.001). Type 2 EGJ with Milan Score < 137 resembled type 1 EGJ (objective GERD in 23.6% vs. 33.2%, *p* = 0.09), and type 2 EGJ with score ≥ 137 resembled type 3 EGJ (objective GERD in 88.2% vs. 78.8%, *p* = 0.11). On ROC analysis, the Milan Score had an area under the curve of 0.858.

**Conclusion:**

While type 2 EGJ includes varying GERD severity, the Milan Score can segregate patients at risk for objective GERD.

## Graphical abstract

The novel manometric parameter Milan Score could be useful in predicting which patients with type 2 EGJ have a high likelihood of objective GERD, therefore discriminating which patients may need escalation of GERD investigation and management.

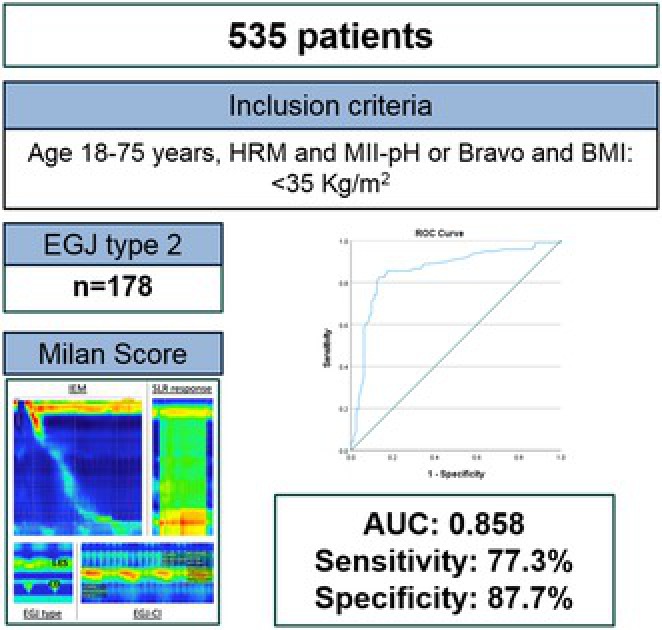

**Summary**

- Patients with type 2 EGJ present two different clinical entities, that can be identified by HRM metrics.
- The novel Milan Score could be useful in predicting which patients with type 2 EGJ have a high likelihood of definitive GERD.

## 1 Introduction

Gastroesophageal reflux disease (GERD) is one of the most commonly diagnosed medical conditions globally, with an estimated prevalence that varies from 5% in Asia to 10%–20% in the Western countries [1, 2, 3]. Objective GERD diagnosis, based on the Lyon Consensus 2.0, relies on evidence of erosive esophagitis on endoscopy (grades B, C, and D according to the Los Angeles classification) or esophageal acid exposure time (AET) > 6% on multichannel intraluminal impedance pH monitoring (MII‐pH) [4, 5]. However, neither of these investigations provides a precise insight into the actual pathophysiology of GERD, but esophagogastric junction (EGJ) barrier function can be elucidated using high‐resolution manometry (HRM), thereby providing pathophysiologic mechanisms leading to GERD. A recent study introduced the Milan Score [6], a novel HRM tool to predict GERD and stratify its severity by integrating four parameters: ineffective esophageal motility (IEM), esophagogastric‐junction contractile integral (EGJ‐CI), EGJ morphology, and straight leg raise (SLR) maneuver. These variables represent the anatomical and physiological factors favoring GERD, including esophageal clearance, esophagogastric‐junction integrity, the presence of hiatal hernia (HH), and the ability of the EGJ to withstand an increase in abdominal pressure without increasing intrathoracic pressure [7, 8, 9].

One of the most important anatomical factors favoring GERD is the presence of a hiatal hernia, and this can be reliably identified using HRM [10]. The Chicago Classification 4.0 characterizes EGJ morphology into three types: type 1, when the LES is superimposed with the crural diaphragm (CD); type 2, when the axial separation between the two entities is < 3 cm; type 3, when the separation is ≥ 3 cm [11]. While it is widely accepted that a type 3 EGJ is a strong predictor of GERD, type 2 represents a gray area with unclear impact on the pathogenesis and severity of the disease.

We hypothesized that in a population of patients with reflux symptoms undergoing a thorough pathophysiologic evaluation, HRM variables and the Milan Score could provide better characterization of risk for objective GERD in patients with EGJ type 2.

## 2 Methods

Consecutive patients with suspected GERD prospectively enrolled in an international multicenter observational study [6] between July 2021 and February 2023 in 13 high‐volume tertiary academic institutions were retrospectively evaluated. Patients aged 18 to 75 years were included if they had undergone HRM and off‐PPI ambulatory reflux monitoring (either wireless or catheter‐based) for GERD symptoms within 2 weeks of each other. Exclusion criteria consisted of BMI > 35 kg/m^2^, history of prior foregut surgery, eosinophilic esophagitis, esophageal achalasia, or other major motility disorders. Objective GERD was diagnosed based on any of the following criteria: AET > 6% on MII‐pH; at least 2 days of AET > 6% on wireless pH monitoring; Los Angeles grades B, C, or D esophagitis, biopsy‐proven Barrett's esophagus, or peptic stricture on endoscopy. GERD was excluded when AET < 4%. In case of inconclusive evidence of GERD (AET 4%–6%), a mean nocturnal basal impedance (MNBI) < 1500 Ω, a total number of reflux episodes > 80/day, or reflux‐symptom association on MII‐pH provided adjunctive evidence for objective GERD according to Lyon 2.0 [4].

The study was approved by our institutional review board and was conducted following the Declaration of Helsinki, and each participating center obtained local institutional review board approval. All patients provided written informed consent, and none of the patients received compensation for participation in the study.

### 2.1 Clinical Characteristics

Demographics, clinical variables (age, sex, BMI, symptom onset, cigarette smoking, primary and secondary symptoms, PPI consumption and response), and results of investigation (hiatal hernia and reflux on barium swallow study, hiatal hernia, esophagitis, Barrett's esophagus, or peptic stricture on upper endoscopy) were retrieved from the patients' clinical records. GERD symptoms were also assessed using validated questionnaires prior to HRM: GERD‐Q [12], GERD Health Related Quality of Life (GERD‐HRQL) [13], and Reflux Symptom Index (RSI) [14].

### 2.2 Esophageal High‐Resolution Manometry

Esophageal HRM was performed following the CCv4.0 standard protocol [11], using a solid‐state catheter with 36 pressure channels spaced at 1‐cm intervals, with each institution using their preferred system. The test was performed by an experienced physician or nurse after overnight fasting. The catheter was positioned transnasally, and an adaptation period of 1 min was observed. After a baseline period of 30 s to allow identification of anatomical landmarks, 10 swallows of 5 mL of water were performed in the primary position (upright or recumbent), followed by five 5 mL swallows in the secondary position. Multiple rapid swallows (MRS) were then performed, administering the patient 5 swallows of 2 mL of water with a 3‐s interval or shorter. Contraction reserve was present on MRS when the ratio between MRS‐DCI and the mean DCI of single swallows was > 1.

Finally, the SLR maneuver was performed in a supine position as previously described [9]. If elevation of one leg was not sufficient to elicit an increase of intra‐abdominal pressure, the patient was asked to perform a double leg raise. SLR was considered effective if there was a 50% increase in intra‐abdominal pressure (IAP) during the maneuver and positive if there was an increase of 11 mmHg in the intra‐esophageal peak pressure from baseline to the maneuver, measured 5 cm above the LES [9].

Swallows were characterized according to CCv4.0, based on distal contractile integral (DCI): ≥ 450 mmHg*cm*s were defined as intact swallows, 100–450 mmHg*cm*s as weak swallows, and ≤ 100 mmHg*cm*s as failed swallows. Both weak and failed swallows were considered ineffective. Ineffective esophageal motility was defined as having > 70% ineffective swallows or ≥ 50% failed swallows.

LES and EGJ were assessed using LES basal pressure, EGJ‐CI, total and intra‐abdominal LES length, and EGJ morphology, segregated into 3 groups based on the relationship between the LES and the CD: type 1 was defined by overlap between LES and CD, type 2 by separation of < 3 cm, and type 3 by separation of ≥ 3 cm.

EGJ‐CI was calculated by enclosing the upper and lower margins of the EGJ in the DCI toolbox for three consecutive respiratory cycles, with the threshold isobaric contour set at pressure. The DCI tool in mmHg s cm was then divided by the duration of the three respiratory cycles, yielding EGJ‐CI units of mmHg*cm [15].

**FIGURE 1:**
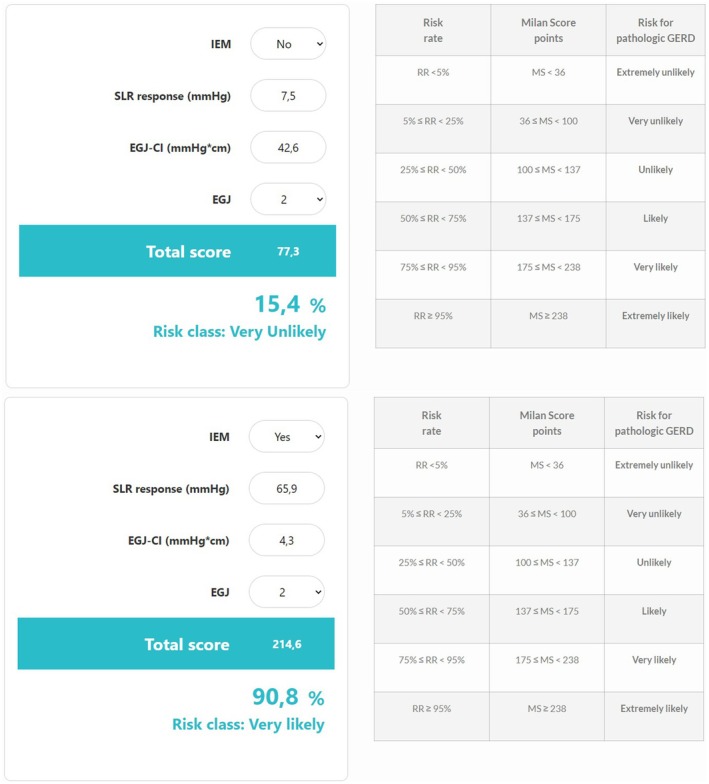
Two examples of Milan Score with risk stratification tables (www.milanscore.com).

The Milan Score is a novel HRM metric that was developed to evaluate the risk of pathological GERD. For each patient, the Milan Score was calculated from the following variables: IEM, SLR maneuver, EGJ‐CI, and EGJ morphology [6]. A validated statistical model assigns a score to each variable. The final Milan Score, determined by the sum of individual scores and calculated by a web and mobile app (www.milanscore.com) serves as a reliable predictor of AET > 6%. Higher scores correspond to a greater degree of disruption in the anti‐reflux barrier function. Risk stratification based on the Milan Score categorizes patients into six distinct risk classes, ranging from extremely unlikely to extremely likely to have objective GERD (Figure 1).

### 2.3 Esophageal Ambulatory pH and pH‐Impedance Monitoring

Reflux monitoring was performed using wireless 96‐h pH monitoring or 24‐h MII‐pH catheter‐based monitoring. The wireless pH capsule was positioned 6 cm above the LES, under endoscopic guidance. The MII‐pH studies were performed by inserting the catheter transnasally after calibration with buffer solutions at pH 4 and 7. The probe was placed with the pH electrode 5 cm above the LES and was left in place for 24 h, during which patients recorded the duration of meals, symptoms, and time spent in the recumbent position.

After completion, all pH and MII‐pH tracings were reviewed by experts at each center, excluding meal periods from the analysis. Recorded data included total, upright, and recumbent AET; number of acid, weakly acid, and weakly alkaline reflux episodes; DeMeester score; bolus exposure time; symptom index (SI); and symptom association probability (SAP) [16]. Additionally, MNBI and PSPW indices were recorded for MII‐pH studies.

AET was considered physiological if < 4%, abnormal when > 6%, and borderline when between 4% and 6%; total, upright, and recumbent AET were assessed separately. According to the Lyon Consensus 2.0, < 40 reflux episodes over 24 h were considered normal, while > 80 were considered pathological [4].

MNBI was assessed during 3 overnight 10‐min time periods on the most distal impedance channel and was considered pathological if < 1500 Ω. PSPW was calculated as the proportion of the reflux events followed by a swallow‐induced peristaltic wave within 30 s, and was abnormal if lower than 50% [17, 18]. Finally, the SI was defined as the number of symptoms preceded by a reflux episode and was positive when > 50%, while the SAP was the overall probability that symptoms were associated with reflux episodes and was positive when > 95%.

### 2.4 Statistical Analysis

Patients were segregated based on EGJ morphology into the three EGJ subtypes. Clinical, HRM, and pH characteristics were compared between the groups. The Milan Score was tested on EGJ type 2 patients to determine the likelihood of a definitive GERD diagnosis based on AET > 6% and endoscopic findings. Patients in the EGJ type 2 group were further divided into subgroups based on whether the Milan score was below the optimal cut‐off of 137, or ≥ 137 which were then compared with EGJ subtypes 1 and 3.

After evaluating normality of continuous variables with the Shapiro–Wilk test, the Kruskal‐Wallis test with Dunn's post hoc analysis was used for continuous variables and the Fisher exact test with Bonferroni's post hoc correction for categorical variables within a non‐normal distribution. One‐way analysis of variance (ANOVA) and chi‐square tests with Bonferroni post hoc correction were used for continuous and categorical variables with normal distribution, as appropriate. Continuous variables were reported using mean ± standard deviation (SD), and categorical variables were reported using numbers and percentages.

A *p* < 0.05 was considered statistically significant. A receiver operating characteristic (ROC) curve was plotted to illustrate the performance of the Milan Score in the prediction of GERD in patients with an EGJ type 2 morphology. All analyses were performed with IBM SPSS Statistics 29 (IBM, Armonk, NY, USA).

## 3 Results

A total of 535 patients (median age 49.9 ± 14.4 years, 46.4% female, BMI 25.8 ± 5.2 kg/m^2^) were included in the final study group. Based on EGJ morphology: 277 (51.8%) had type 1 EGJ, 178 (33.3%) had type 2 EGJ, and 80 (15.0%) had type 3 EGJ. Demographics, clinical, and endoscopic characteristics of patients are described in Table 1.

**TABLE 1:** Demographics, clinical and endoscopic characteristics according to EGJ type.

	EGJ type 1 (*n* = 277)	EGJ type 2 (*n* = 178)	EGJ type 3 (*n* = 80)	*p*
Age (years), mean (SD)	**47.2 (13.7)** ^**a**^	52.9 (14.7)	52.5 (14.3)	** << 0.001**
Gender (female), *n* (%)	132 (47.6)	78 (43.8)	38 (47.5)	0.749
BMI (kg/m^2^), mean (SD)	**25.3 (5.5)** ^**a**^	25.9 (4.6)	**27.3 (4.9)** ^**a**^	**0.010**
Smoking, *n* (%)	39 (14.1)	20 (11.2)	**26 (32.5)** ^**a**^	**0.001**
Symptom duration (months), mean (SD)	55.4 (58.4)	58.9 (72.1)	45.6 (46.8)	0.316
Symptoms, *n* (%)				
Heartburn	139 (50.2)	99 (55.9)	**61 (76.3)** ^**a**^	** < 0.001**
Regurgitation	45 (16.2)	31 (17.4)	14 (17.5)	0.934
Epigastric pain	33 (11.9)	13 (7.3)	10 (12.5)	0.238
Atypical symptoms	61 (22.0)	35 (19.7)	13 (16.3)	0.639
PPI use, *n* (%)	**185 (66.8)** ^**a**^	133 (74.7)	**70 (87.5)** ^**a**^	**0.008**
Response to PPI, *n* (%)				**0.048**
No response	**98 (35.3)** ^**a**^	40 (22.5)	17 (21.2)	
Partial response	**124 (44.9)** ^**a**^	98 (55.0)	**48 (60.0)** ^**a**^	
Full benefit	55 (19.8)	40 (22.5)	15 (18.8)	
GERD‐Q A score, mean (SD)	7.6 (3.8)	7.3 (3.7)	6.9 (3.4)	0.388
GERD‐Q B score, mean (SD)	2.5 (2.0)	2.3 (2.1)	2.2 (2.0)	0.500
GERD‐HRQL score, mean (SD)	15.6 (9.8)	15.6 (9.5)	18.9 (11.1)	0.063
RSI score, mean (SD)	12.7 (9.5)	14.2 (9.6)	12.4 (10.3)	0.305
Endoscopy*				
Hiatal hernia, *n* (%)	**121 (48.6)** ^**a**^	**107 (68.2)** ^**a**^	**71 (91.0)** ^**a**^	**< 0.001**
Hiatal hernia size (cm), mean (SD)	0.5 (0.3)^**a**^	1.4 (0.6)	**3.3 (1.3)** ^**a**^	**0.017**
Esophagitis, *n* (%)				**< 0.001**
No esophagitis	**187 (74.8)** ^**a**^	100 (64.1)	**43 (55.8)** ^**a**^	
Grade A	45 (18.0)	27 (17.3)	10 (13.0)	
Grade B	**13 (5.2)** ^**a**^	21 (13.5)	16 (20.8)	
Grade C	1 (0.4)	3 (1.9)	**7 (9.1)** ^**a**^	
Grade D	4 (1.6)	5 (3.2)	1 (1.9)	
Barrett's esophagus	10 (5.6)	14 (8.9)	3 (3.8)	0.085

Abbreviations: BMI, body mass index; *n*, number; PPI, proton pump inhibitors; SD, standard deviation. Bold means statistically significant.

^a^ Statistically significant difference on post hoc analysis.

* 484 patients underwent endoscopy (249 in EGJ type 1, 157 in EGJ type 2, 78 in EGJ type 3).

Patients with type 1 EGJ were significantly younger and had a lower BMI compared to patients with type 3 EGJ. Heartburn was more frequent in the type 3 EGJ group, while proportions of other symptoms and clinical scores did not differ among the three EGJ subtypes. PPI use was more frequent in type 3 EGJ, but patients with type 1 EGJ had a higher rate of non‐responders (35.3%) compared to type 2 EGJ (22.5%) and type 3 (21.2%) (*p* = 0.008). Among the 484 patients who underwent upper GI endoscopy, patients with type 3 EGJ had an overall higher rate of esophagitis compared to type 1 EGJ (44.2% vs. 25.2%), and grade C esophagitis was significantly more represented.

Table 2 shows HRM and pH study data of included patients. HRM showed significant differences between the EGJ subtypes in all parameters analyzed: patients with type 1 EGJ had longer total and intra‐abdominal LES, higher EGJ‐CI, lower rates of IEM and positive SLR maneuver, and consequently, lower median Milan score and GERD risk compared to the other two EGJ subtypes (*p* < 0.001 for all). In contrast, patients with type 2 EGJ were similar to type 3 EGJ patients in terms of LES length and rates of IEM but differed from the other groups in EGJ‐CI values, rates of positive SLR maneuver, Milan score, and GERD risk. On esophageal pH monitoring, 45.5% of patients in the type 2 EGJ group had AET > 6%, significantly higher than type 1 EGJ (26%, *p* < 0.001) and lower than type 3 EGJ (77.5%, p < 0.001). Patients with type 3 EGJ had a higher rate of patients with more than 80 total reflux episodes (27%), compared with patients with type 1 EGJ (9.1%, *p* < 0.001). Rates of MNBI < 1500 Ω were different among the groups (21.8% in type 1 EGJ, 42.6% in type 2 EGJ and 59.1% in type 3 EGJ, *p* < 0.001).

**TABLE 2:** HRM and pH data according to EGJ type.

	EGJ type 1 (*n* = 277)	EGJ type 2 (*n* = 178)	EGJ type 3 (*n* = 80)	*p*
High‐resolution manometry				
Hiatal hernia, *n* (%)	**0 (0)** ^**a**^	178 (100)	80 (100)	**< 0.001**
LES total length (cm), mean (SD)	**2.3 (0.7)** ^**a**^	2.0 (0.6)	1.9 (0.6)	**< 0.001**
LES intra‐abdominal length (cm), mean (SD)	**0.9 (0.8)** ^**a**^	0.0 (0.0)	0.0 (0.0)	**< 0.001**
EGJ‐CI (mmHg*cm), mean (SD)	**52.5 (31.0)** ^**a**^	**40.8 (32.5)** ^**a**^	**21.6 (18.7)** ^**a**^	**< 0.001**
Patients with IEM, *n* (%)	**38 (13.7)** ^**a**^	42 (23.6)	25 (31.3)	**0.001**
Positive SLR maneuver, *n* (%)	**89 (32.1)** ^**a**^	**85 (47.8)** ^**a**^	**56 (70.0)** ^**a**^	**< 0.001**
Milan score, mean (SD)	**86.5 (51.1)** ^**a**^	**131.9 (54.4)** ^**a**^	**184.4 (49.2)** ^**a**^	**< 0.001**
% GERD risk Milan score, mean (SD)	**26.5 (26.9)** ^**a**^	**47.8 (31.9)** ^**a**^	**73.3 (26.6)** ^**a**^	**< 0.001**
Esophageal pH study				
AET (%), mean (SD)	**5.1 (7.3)** ^**a**^	**8.3 (11.1)** ^**a**^	**12.2 (11.3)** ^**a**^	**< 0.001**
4% < AET < 6%, *n* (%)	35 (12.6)	24 (11.8)	6 (7.5)	0.447
AET > 6%, *n* (%)	**72 (26.0)** ^**a**^	**81 (45.5)** ^**a**^	**62 (77.5)** ^**a**^	**< 0.001**
DeMeester score, mean (SD)	**21.7 (30.3)** ^**a**^	34.6 (44.8)	45.3 (39.4)	**< 0.001**
Patients with SI > 50%, *n* (%)	101 (36.5)	81 (45.5)	36 (45.0)	0.112
Patients with SAP > 95%, *n* (%)	83 (30.0)	65 (36.5)	32 (40.0)	0.151
Total reflux episodes, mean (SD)	41.7 (33.5)	48.9 (31.2)	**66.1 (47.2)** ^**a**^	**< 0.001**
Reflux episodes > 80, *n* (%)	**24 (9.1)** ^**a**^	26 (15.5)	**20 (27.0)** ^**a**^	**< 0.001**
MNBI < 1500 Ω, *n* (%)^b^	**36 (21.8)** ^**a**^	**46 (42.6)** ^**a**^	**39 (59.1)** ^**a**^	**< 0.001**
PSPW < 50%, *n* (%)^b^	**54 (32.7)** ^**a**^	**57 (52.7)** ^**a**^	**51 (77.2)** ^**a**^	**< 0.001**

Abbreviations: CRC, colorectal cancer; *n*, number; SD, standard deviation. Bold means statistically significant.

^a^ Statistically significant difference on post hoc analysis.

^b^ 339 patients who underwent multichannel intraluminal impedance‐pH study (165 in EGJ type 1, 108 in EGJ type 2, 66 in EGJ type 3).

**FIGURE 2:**
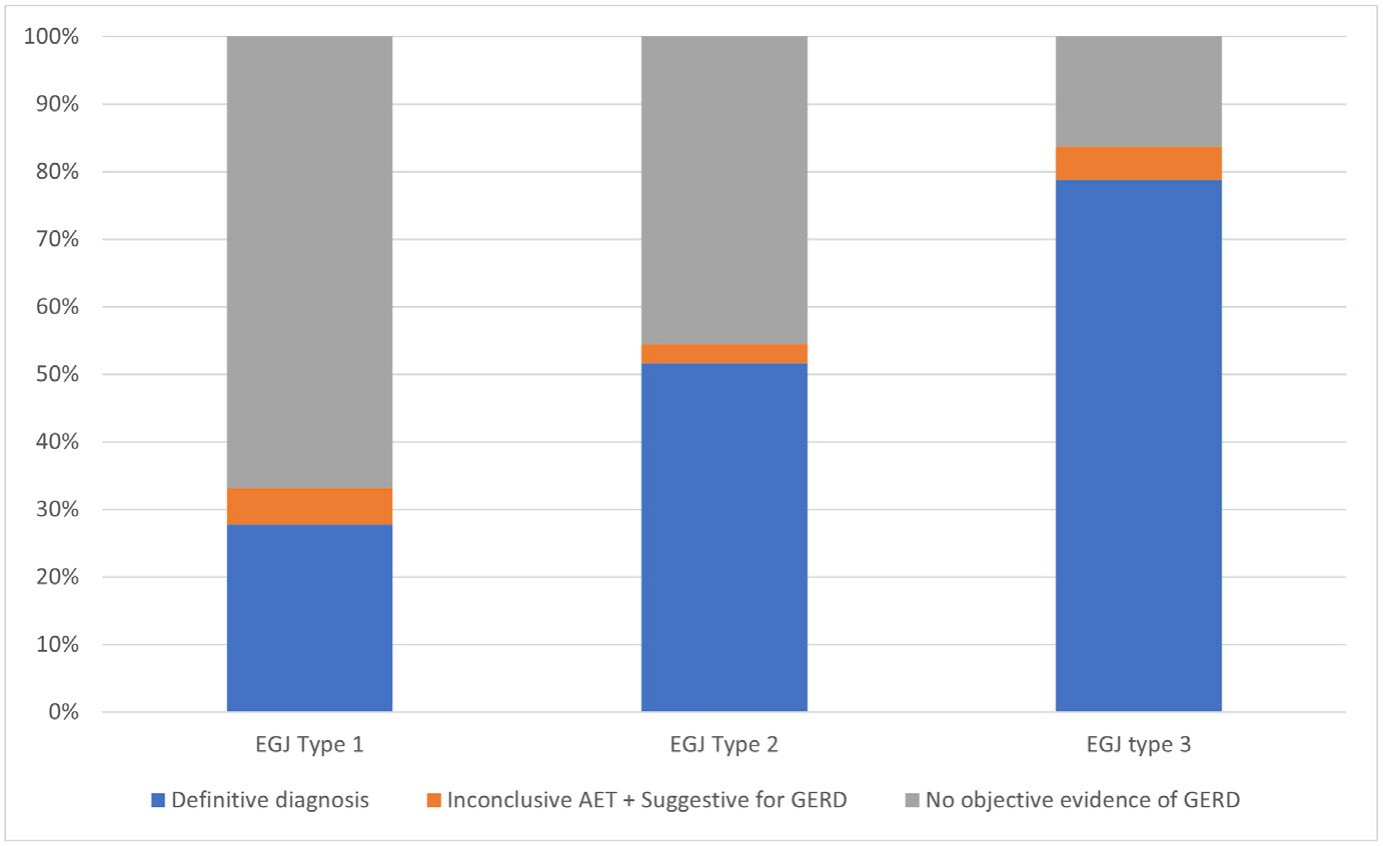
Rates of patients with objective findings of GERD in different EGJ types.

Overall, 83.8% of patients in the type 3 EGJ group had an objective diagnosis of GERD, as 78.8% had AET > 6% or grade B, C, or D esophagitis, and a further 5% had a borderline AET plus adjunctive evidence of GERD. Using the same criteria, in the EGJ 2 group, 54.5% of patients had an objective diagnosis of GERD (51.7% definitive evidence and 2.8% adjunctive evidence), and in type 1 EGJ, 33.2% (27.8% definitive evidence and 5.4% adjunctive evidence) (Figure 2).

### 3.1 Application of the Milan Score

**FIGURE 3:**
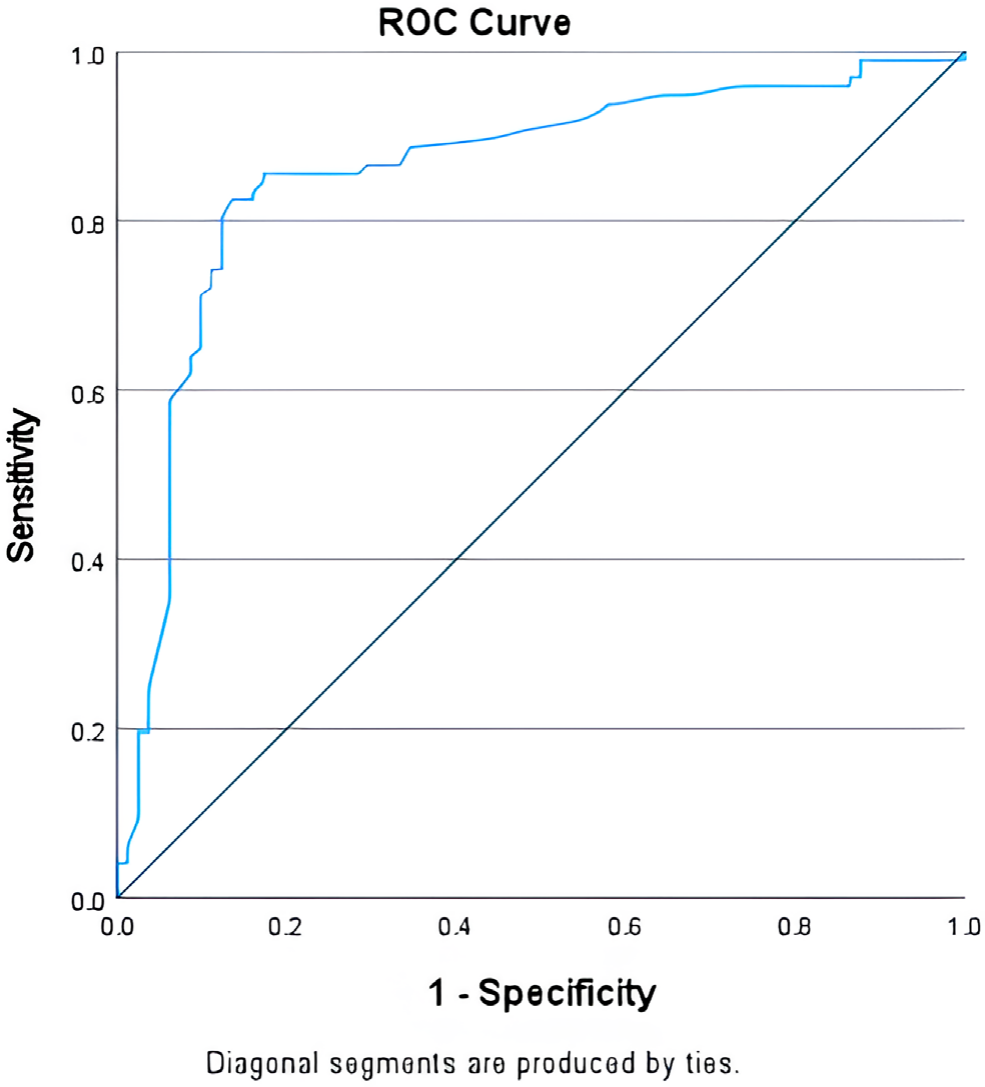
ROC curve illustrating the performance of the Milan Score in the prediction of patients with objective GERD in EGJ type 2 morphology.

**FIGURE 4:**
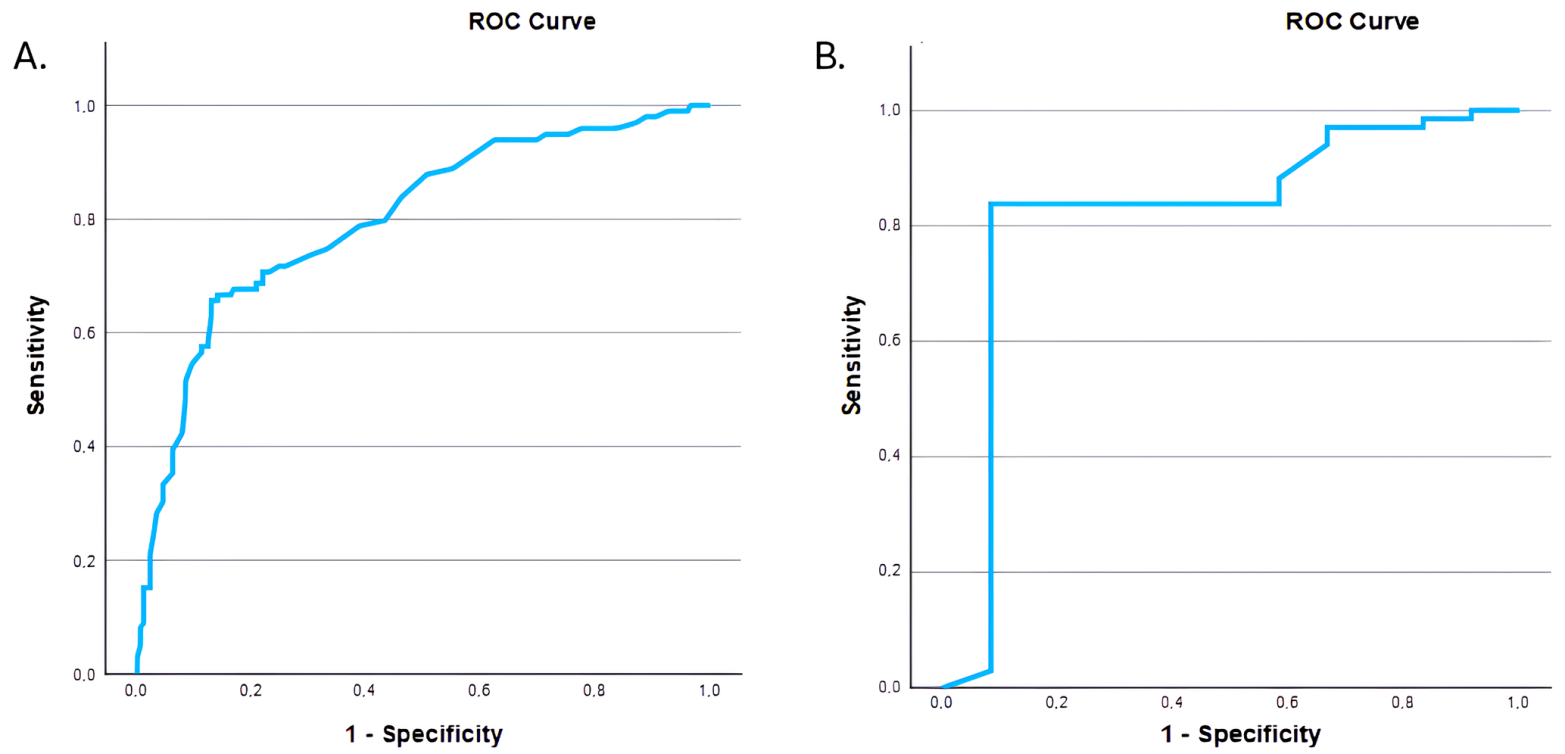
ROC curve illustrating the performance of the Milan Score in the prediction of patients with objective GERD in EGJ type 1 (A) and type 3 (B) morphology.

When the Milan Score was applied to discriminate patients with objective GERD within the type 2 EGJ group, the area under the curve (AUC) of the ROC analysis was 0.858 (Figure 3). At the optimal threshold to identify pathologic GERD of 137 points, sensitivity was 77.3%, and specificity was 87.7%. When the Milan Score at the threshold of 137 was tested in type 1 and type 3 EGJ groups, we found an AUC of 0.801 for type 1 (63.6% sensitivity and 86.5% specificity) (Figure 4a) and an AUC of 0.824 for type 3 (82.4% sensitivity and 91.7% specificity) (Figure 4b). The same analysis was performed for positive SLR, and the AUC of the ROC analysis was 0.811 (Figure S1).

The subgroups of patients in the EGJ type 2 with Milan Score < 137 versus ≥ 137 were then compared to type 1 and 3 EGJ (Table 3). Patients with a Milan Score ≥ 137 were similar to patients with type 3 EGJ in terms of mean AET, DeMeester Score, > 80 reflux episodes, MNBI < 1500 Ω, and PSPW < 50%. In contrast, patients with Milan Score < 137 had similar pH characteristics as type 1 EGJ, differing significantly from patients with Milan Score ≥ 137. The rate of pathologic GERD was similar between type 1 and type 2 with Milan Score < 137 (33.2% vs. 23.6%, *p* = 0.09) and between type 3 and type 2 with Milan Score ≥ 137 (77.3% vs. 88.2%, *p* = 0.11), and the former two groups collectively were significantly different from the latter two groups (30.8% vs. 83.0% respectively, *p* > 0.001).

**TABLE 3:** HRM and pH data stratified by EGJ type. Patients with EGJ type 2 have been divided in subgroups of patients with Milan score < 137 and > 137.

	EGJ type 1 (*n* = 277)	EGJ type with MS < 137 (*n* = 93)	EGJ type with MS ≥ 137 (*n* = 85)	EGJ type 3 (*n* = 80)	*p*
High‐resolution manometry					
Hiatal hernia, *n* (%)	21 (7.7)^a^	75 (82.4)^b^	69 (83.1)^b^	77 (97.5)^c^	**< 0.001**
LES total length (cm), mean (SD)	2.3 (0.7)^a^	2.1 (0.6)^b^	2.0 (0.6)^b^	2.0 (0.6)^b^	**< 0.001**
LES intra‐abdominal length (cm), mean (SD)	0.9 (0.7)^a^	0.1 (0.4)^b^	0.1 (0.4)^b^	0.1 (0.7)^b^	**< 0.001**
EGJ‐CI (mmHg*cm), mean (SD)	52.5 (31.0)^a^	47.4 (35.7)^a^	33.6 (26.9)^b^	21.6 (18.7)^b^	**< 0.001**
Patients with IEM, *n* (%)	38 (13.7)^a^	21 (22.6)^a,b^	21 (24.0)^a,b^	25 (31.3)^b^	**0.002**
Esophageal pH study					
AET (%), mean (SD)	5.1 (7.3)^a^	3.9 (5.8)^a^	13.2 (13.3)^b^	12.2 (11.1)^b^	**< 0.001**
4% < AET < 6%, *n* (%)	35 (12.6)	14 (15.1)	7 (8.2)	6 (7.5)	0.305
AET > 6%, *n* (%)	72 (26.0)^a^	12 (12.9)^a^	69 (81.2)^b^	62 (77.5)^b^	**< 0.001**
Pathologic GERD, *n* (%)	92 (33.2)^a^	22 (23.6)^a^	75 (88.2)^b^	62 (77.3)^b^	**< 0.001**
DeMeester score, mean (SD)	21.7 (30.3)^a^	15.7 (23.2)^a^	55.0 (53.0)^b^	45.3 (39.5)^b^	**< 0.001**
Patients with SI > 50%, n (%)	101 (36.5)^a^	33 (35.5)^a^	48 (56.5)^b^	36 (45.0)^a,b^	**0.006**
Patients with SAP > 95%, *n* (%)	83 (30.0)	30 (32.3)	35 (41.2)	32 (40.0)	0.147
Total reflux > 80, *n* (%)	37 (13.4)^a^	12 (12.9)^a^	24 (28.2)^b^	25 (31.3)^b^	**< 0.001**
MNBI < 1500 Ω, *n* (%)*	36 (21.8)^a^	11 (20.0)^a^	35 (66.0)^b^	39 (59.1)^b^	**< 0.001**
PSPW < 50%, *n* (%)*	54 (32.7)^a^	18 (32.3)^a^	39 (75.8)^b^	51 (77.2)^b^	**< 0.001**

*Note:* Same letters in superscript indicate statistically significant similarity on Bonferroni post hoc analysis. Bold means statistically significant.

Abbreviations: *n*, number; SD, standard deviation.

* 339 patients who underwent multichannel intraluminal impedance‐pH study.

## 4 Discussion

In this study, we demonstrate that the Milan Score is able to discriminate patients with pathologic GERD on esophageal testing within patients with type 2 EGJ. While current literature has shown that type 3 EGJ is strongly associated with objective GERD evidence, type 2 EGJ still represents a clinical conundrum, with an unclear impact in the pathophysiology of GERD [19, 20]. In contrast, type 3 EGJ has a higher rate of hiatal hernia on both endoscopy and HRM. Our clinical analysis supports the fact that type 1 and 3 EGJs are clearly distinct clinical entities, while type 2 EGJ stands in between with a trend towards type 3 in certain variables (i.e., PPI nonresponders). In addition to the Milan Score, other HRM variables related to GERD provided further insights on type 2 EGJ. The likelihood of IEM was similar to type 3 EGJ, an important finding, given the impact that IEM plays in GERD pathophysiology [21, 22]. We conclude that HRM and calculation of the Milan Score has value in determining which type 2 EGJ patients has enough EGJ barrier disruption to warrant investigation and management of abnormal reflux burden.

When considering the SLR maneuver response and EGJ‐CI, type 2 EGJ was significantly different compared to both type 1 and 3 EGJ, with findings that place type 2 EGJ in between these two subtypes. Reflux monitoring demonstrated a stepwise increase in mean AET from type 1 to 3 EGJ, further affirming the ability of the CCv4.0 EGJ morphology subtypes to stratify GERD patients. These results confirm that type 2 EGJ is an intermediate entity between the predominantly intact barrier function seen with type 1 EGJ (pathologic GERD: 33.2%) and those with higher prevalence of GERD with type 3 EGJ (83.8%) (Figure 2).

We hypothesized that the wide “gray area” of type 2 EGJ could contain two very different patient phenotypes, with clinical and physiological characteristics similar to those with either type 1 or 3 EGJ. The recently validated Milan Score [6] was an optimal tool to test this hypothesis since the Milan Score relates to esophageal pathophysiology in GERD, particularly EGJ barrier function. With these assumptions, the Milan performed very well with an AUC of 0.858 on ROC analysis in predicting objective GERD, and the optimal value that predicted pathologic GERD within type 2 EGJ was 137. Of note, while SLR alone showed good predictive ability (AUC 0.811), the composite assessment provided by the Milan Score demonstrated superior diagnostic accuracy (AUC 0.858) using the threshold of 137 points, confirming the value of integrating multiple pathophysiologic parameters for the assessment of the EGJ. In fact, in our validation studies, such value corresponds to a risk rate of GERD of 50%, which separates the Milan Score categories “unlikely” and “likely.”

We acknowledge that using EGJ morphology both as a component of the Milan Score and for patient stratification could appear methodologically circular. However, since all patients in our type 2 EGJ analysis had the same morphology classification, we were able to demonstrate that the other components of the Milan Score (i.e., IEM, EGJ‐CI, and SLR) can be used to identify different phenotypes within the type 2 EGJ group.

Using the Milan Score, patients with type 2 EGJ could be segregated into two subgroups, one with Milan Score < 137, which resembled patients with type 1 EGJ, and the other with Milan Score ≥ 137 that shared the same pathophysiologic characteristics with type 3 EGJ. Without denying the anatomical uniqueness of type 2 EGJ, we hypothesize that the clinical and physiological characteristics of these two groups could resemble those of patients with absent or pronounced LES and crura separation, as in type 1 and 3 EGJ, and, even more interestingly, they could more precisely predict pathologic GERD. In fact, patients that share the same anatomic characteristics (LES‐CD separation < 3 cm) appear to have at least two different pathophysiologic consequences identified using the Milan Score, one resembling the less refluxogenic type 1 EGJ and the other resembling the highly reflux‐prone type 3 EGJ. The reason for this distinction is still unknown, and further studies are needed to scrutinize different mechanisms, including the width of the hiatus, persistent vs. intermittent hiatal hernia, or different classifications of EGJ based on the location of the pressure inversion point (PIP) as described by Akimoto et al. [23].

The main strength of the present study is its thorough characterization of patients who underwent a complete pathophysiological and clinical evaluation of not just EGJ barrier function morphology but also esophageal body motor function and reflux burden. Other strengths consist of the use of strict HRM and reflux monitoring protocols, as well as validated questionnaires that allowed standardized assessment, the multicenter setting, and the inclusion of real‐life data, which has enhanced our understanding of the complexity of GERD.

This study has some limitations: all procedures were performed in high‐volume centers by expert operators, potentially limiting the generalizability of the findings. We relied on individual centers and their experts to analyze HRM and reflux‐monitoring studies, which could have introduced inter‐observer bias, especially in categorizing patients into individual EGJ morphology subgroups. Furthermore, the use of new diagnostic tools such as SLR, which have not yet been widely accepted or included in official guidelines and classification, could further impact the incorporation of our results on a large scale. In this regard, although the SLR maneuver has proven utility in the assessment of reflux burden, it still has to be fully investigated. It is possible that, in a small proportion of patients, the chest straining during the elevation of the legs could produce an increased esophageal pressure without an actual EGJ incompetence (false positive). However, normal breathing during the maneuver could mitigate this issue. In this study, we did not record such data, but we plan to do so in future studies on SLR.

While our study used an esophageal pressure increase of 11 mmHg to define SLR positivity [9], we acknowledge that this metric requires further validation. Future studies should consider correlating the esophageal pressure increase during SLR with other objective measures of EGJ competence and potentially explore alternative thresholds for defining a positive response.

Finally, another significant limitation of our study is that we used standard HRM catheters rather than combined high‐resolution manometry‐impedance (HRMZ) catheters. HRMZ would have allowed direct correlation between manometric findings and reflux episodes, potentially providing stronger validation of the Milan Score's predictive value. This limitation highlights the need for further validation studies of the Milan Score using HRMZ technology to directly link manometric parameters with reflux events. Until such validation is completed, the Milan Score should be considered a promising but still‐evolving tool for GERD prediction.

In conclusion, we performed thorough clinical and pathophysiologic characterization of patients with type 2 EGJ and identified two different clinical entities within the group, based on HRM metrics. A Milan Score of 137 could be a useful threshold in predicting which patients with type 2 EGJ have a high likelihood of objective GERD, which may allow distinction of which patients may need escalation of GERD investigation and management.

## Author Contributions

Davide Ferrari, Edoardo Savarino, C. Prakash Gyawali, Nicola De Bortoli, Stefano Siboni, and Marco Sozzi, were involved in conception of the study, acquisition of the data, analysis and interpretation of the data, drafting the paper, and approving the final manuscript. Pierfrancesco Visaggi, Ivan Kristo, Salvatore Tolone, Benjamin D. Rogers, Anthony Hobson, Jordan Haworth, Yeong Yeh Lee, Brian E. Louie, Takahiro Masuda, Megan L. Ivy, Pamela Milito, Erica Centorrino, Dimitrios Theodorou, Tania Triantafyllou, Andrea Pasta, Francesco Calabrese, Vincent Tee, Lorenzo Cusmai, Roberto Penagini, Emanuele Asti, and Marina Coletta were involved in acquisition of the data, drafting the paper, and approving the final manuscript.

## Conflicts of Interest

The authors declare no conflicts of interest.

## Supporting information


**Figure S1.** ROC curve illustrating the performance of the positive Straight Leg Raise Maneuver in the prediction of patients with objective GERD in EGJ type 2 morphology.

## Data Availability

The data that support the findings of this study are available on request from the corresponding author. The data are not publicly available due to privacy or ethical restrictions.
