# Supplementary figures and images for: The Milan Score Predicts Objective Gastroesophageal Reflux Disease in Patients With Type 2 Esophagogastric Junction
Source: Neurogastroenterol Motil. 2025 Jan 6;38(1):e14987. doi: 10.1111/nmo.14987 (PMC12849986; doi:10.1111/nmo.14987)

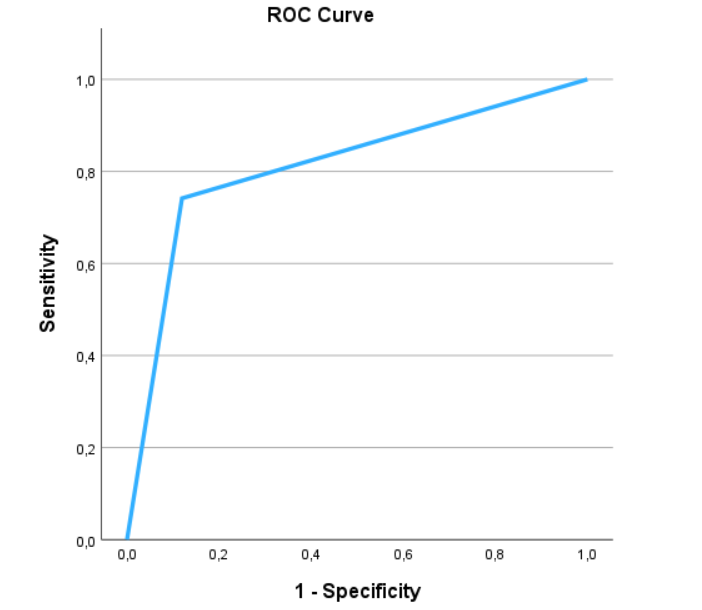

Supplement: Supplementary file 1 — Figure S1. ROC curve illustrating the performance of the positive Straight Leg Raise Maneuver in the prediction of patients with objective GERD in EGJ type 2 morphology. [file NMO-38-e14987-s001.png]
